# Development of an atherosclerosis rabbit model to evaluate the hemodynamic impact of extracorporeal circulation

**DOI:** 10.1002/ame2.12556

**Published:** 2025-02-05

**Authors:** Anna Kathrin Assmann, Jan Buschmann, Sinje Reimers, Aleyna Karakas, Elvira Weber, Hug Aubin, Artur Lichtenberg, Alexander Assmann

**Affiliations:** ^1^ Department of Cardiac Surgery and CURE 3D Lab, Medical Faculty Heinrich Heine University, University Hospital Düsseldorf Düsseldorf Germany; ^2^ CURE 3D Lab, Medical Faculty Heinrich Heine University Düsseldorf Germany; ^3^ Cardiovascular Research Institute Duesseldorf (CARID), Medical Faculty Heinrich‐Heine University Düsseldorf Germany

**Keywords:** animal model, atherosclerosis, calcification, extracorporeal circulation, inflammation, plaques

## Abstract

**Background:**

Aortic atherosclerosis increases the risk of embolic events under extracorporeal circulation (ECC). To evaluate the hemodynamic impact of ECC on atheromatous plaques, an atherosclerosis animal model, which is also eligible for ECC, is required.

**Methods:**

Twenty‐nine New Zealand White rabbits received a pro‐atherosclerotic diet (group diet, *n* = 10), a pro‐atherosclerotic diet and additional intraaortic balloon insufflation injury (group BI, *n* = 9), or served as controls (*n* = 10). After 3 or 6 months, aortic explants were analyzed by (immuno‐)histology and RT‐PCR.

**Results:**

Blood serum analyses revealed increased cholesterol‐levels in groups diet and BI compared to controls (3 months: *p* = 0.03 each, 6 months: *p* < 0.0001 each). Aortic inflammatory infiltration was significantly enhanced in groups diet (CD3 at 3 months: *p* < 0.0001, 6 months: *p* = 0.02; CD68 at 3 months: *p* = 0.01) and BI (CD3 at 3 months: *p* < 0.0001, 6 months: *p* = 0.03; CD68 at 3 months: *p* = 0.04, 6 months: *p* = 0.02). Increased intima hyperplasia occurred in both groups (*p* < 0.0001 each). Macroscopic analyses after 3 and 6 months showed ubiquitous lumen‐narrowing aortic plaques. Calcification of the intima and media was increased in groups diet (intima: *p* < 0.0001 at 3 and 6 months; media at 3 months: *p* < 0.0001, 6 months: *p* = 0.01) and BI (intima: *p* < 0.0001 at 3 and 6 months; media at 3 months: *p* < 0.0001, 6 months: *p* = 0.02). Extensive lipid accumulation was found in the intima in both treatment groups (*p* < 0.0001 each).

**Conclusions:**

A rabbit model with high aortic calcific plaque burden—diet‐induced with no implicit need of an additional intimal injury by an intraaortic balloon insufflation due to comparable outcome—exhibiting multiple pathophysiological aspects of human atherosclerosis has been designed and thoroughly characterized. It is suitable for use in future studies on the interaction between atherosclerotic plaques and the arterial blood flow under ECC.

## Graphical abstract

We have developed a model of diet‐induced atherosclerosis in rabbits with typical arterial plaque formations exhibiting multiple pathophysiological mechanisms of human atherosclerosis. The model is suitable for studies on the interaction between atherosclerotic plaques and arterial blood flow, and hemodynamic changes under ECC in particular.

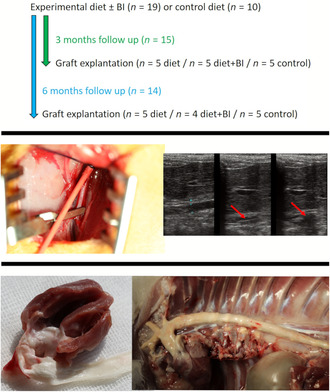

## 1 INTRODUCTION

Extracorporeal circulation (ECC) is routinely used during cardiac surgery as well as for mechanical circulatory support (MCS) due to cardiac or pulmonary failure.^1^ In Germany alone, more than 65 000 heart surgery procedures are conducted with ECC per year.^2^ Even though the use and development of ECC has existed for more than 70 years, there is no consensus yet on the ideal cannulation sites. The choice of the arterial cannulation site (ascending aorta versus axillary or femoral artery) results in either antegrade or non‐physiological retrograde aortic perfusion. Furthermore, the jet stream from the arterial cannula exerts high stress on the arterial wall. In atherosclerotic patients, which represent the majority of cardiosurgical and MCS patients, these changes in hemodynamics may contribute to the mobilization and embolization of atherosclerotic plaques.^3^, ^4^, ^5^, ^6^ Since knowledge on the risk of complications in dependency on different cannulation strategies would influence the clinical cannulation practice, research on this topic is necessary.

In order to examine the interactions between ECC hemodynamics and atherosclerotic plaques, a suitable animal model is required. We have recently established a magnetic resonance imaging‐compatible small animal model under extracorporeal circulation in wildtype rabbits that allows for real‐time blood flow profile measurements during different ECC scenarios.^7^ In addition to that, we have previously developed a rat model of calcifying cardiovascular degeneration.^8^ However, the anatomy of rats is too small to allow for in‐depth studies on arterial blood flow profiles. Furthermore, the predominantly vitamin D‐induced calcification in this model mimics human media arteriosclerosis rather than atherosclerosis. In this context, it should be noted that rats and most other laboratory animals, unlike humans, are not prone to atherosclerosis.^9^ Rabbits represent an exception to this rule, which makes this species attractive for atherosclerosis model development.

Pioneer studies on the induction of cardiovascular degeneration have focused on the role of cholesterol in atherosclerosis^10^ or the pathophysiological interaction between human atherosclerosis and lipid metabolism disorders, particularly addressing the LDL receptor and apolipoprotein E.^11^ The purpose of these studies was to understand the underlying mechanisms of atherosclerosis in order to find an approach to attenuate or even inhibit the initiation or progression of atherosclerosis, which is a major determinant of death worldwide. The impact of pro‐atherosclerotic metabolic conditions on the development of aortic valve stenosis has also been analyzed in rabbit models. Aortic valve stenosis is the most frequent heart valve disease requiring treatment in the western world, and approximately 3%–5% of the population suffer from severe aortic valve stenosis. Therefore, research on the prevention of atherosclerosis as well as on the avoidance of treatment‐associated complications in atherosclerotic patients is of utmost importance.

The aim of our study is the development and in‐depth characterization of an atherosclerosis rabbit model that allows for examination of the impact of different arterial cannulation sites and ECC‐induced hemodynamics on aortic endothelial injury and the mobilization of atherosclerotic plaques. Following these targets, and thereby refining previous research on rabbit models, our project focuses on how the generation of macroscopically evident atherosclerotic plaques changes the geometry of the inner surface of arterial walls, as well as on the profound morphological characterization of vascular wall remodeling.

## 2 METHODS

### 2.1 Animals

Male New Zealand White rabbits (*n* = 29; 2500 g) were obtained from Charles River (Sulzfeld, Germany). Every experiment was conducted according to the *Guide for the Care and Use of Laboratory Animals* and approved by the state animal care committee (reference number 81‐02.04.2020.A383).

**FIGURE 1:**
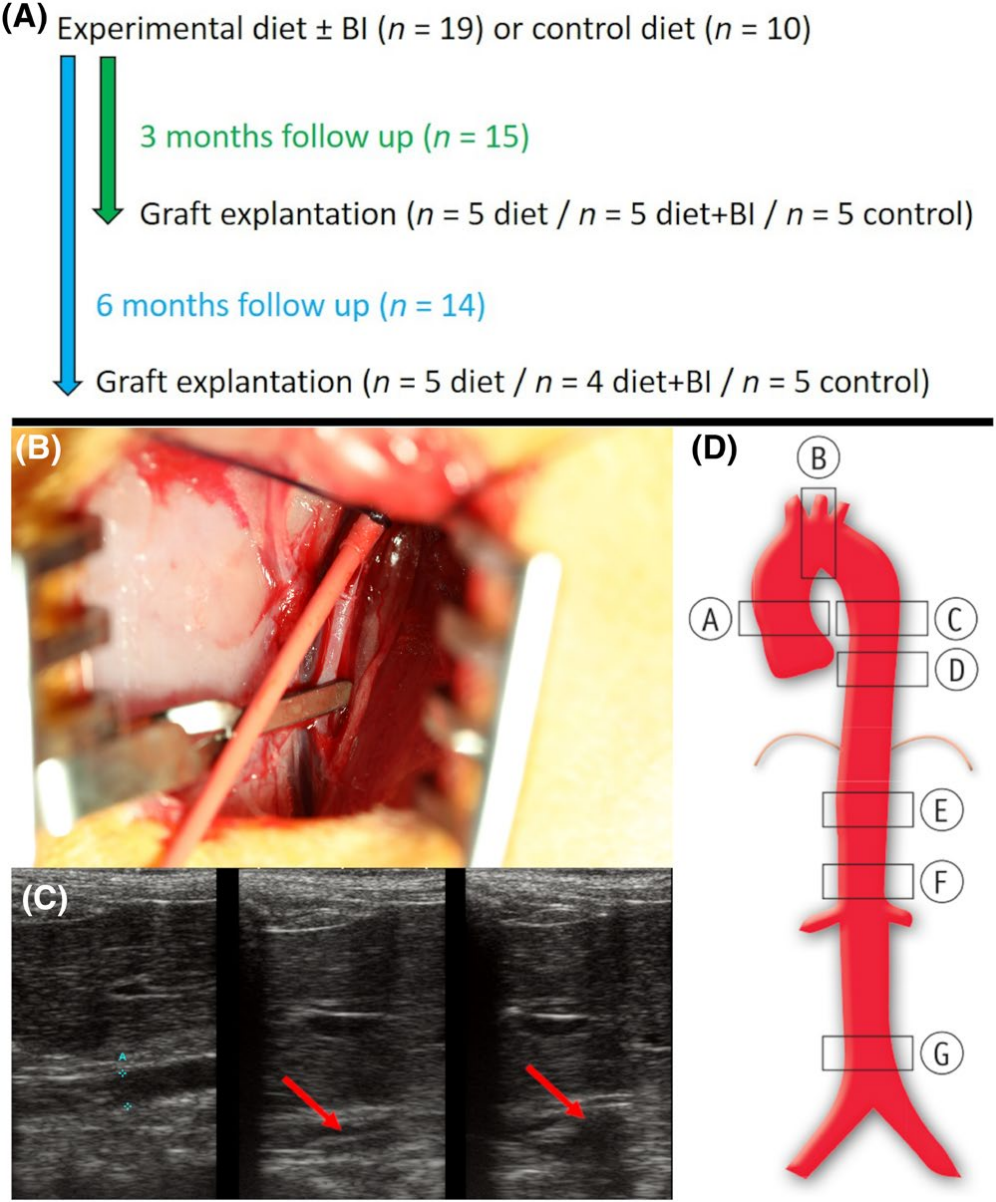
Experimental setup. (A) Experimental timeline. (B) Insertion of the Fogarty catheter (red) into the femoral artery. (C) Insufflation of the balloon (red arrow) to injure the aortic wall under sonographic control. (D) Predefined aortic regions for further analyses: A, ascending aorta; B, aortic arch; C, proximal descending aorta; D, distal descending aorta; E, proximal abdominal aorta; F, suprarenal abdominal aorta; G, infrarenal abdominal aorta. BI, balloon insufflation.

All rabbits were fed ad libitum with standard chow or an experimental diet for 3 or 6 months. In group diet (*n* = 10), rabbits received a pro‐atherosclerotic diet with 0.3% cholesterol and 3% coconut oil (Ssniff SMKV2333, Ssniff GmbH, Soest, Germany), while group BI received pro‐atherosclerotic diet and additional intraaortic balloon insufflation (*n* = 9). Rabbits on standard chow served as controls (*n* = 10) (Figure 1A).

Only male rabbits were used to exclude hormonal fluctuations that might have an impact on the formation of atherosclerotic lesions or the hemodynamics during projected ECC.

### 2.2 Intraaortic balloon insufflation

For intraaortic balloon insufflation, rabbits were orally intubated and anesthetized with 1%–1.5% isoflurane. After an inguinal incision, the femoral artery was exposed and temporarily ligated to perform a vasotomy. A 4F Fogarty‐catheter was retrogradely inserted towards the aorta (Figure 1B). Under echocardiographic control the catheter was insufflated with a defined volume of 0.4 mL in four different positions—in the proximal and distal descending aorta, in the proximal abdominal aorta and in the suprarenal abdominal aorta—to injure the aortic wall (Figure 1C). After extraction of the catheter, the femoral artery was reconstructed with an 8‐0 polypropylene suture, so that an adequate perfusion of the lower limb was guaranteed.

Hereinafter, the rabbits were fed with a pro‐atherosclerotic diet with 0.3% cholesterol and 3% coconut oil for 3 (*n* = 5) or 6 months (*n* = 4).

### 2.3 Explantation of the aorta

After 3 (*n* = 15) or 6 months (*n* = 14), the aorta was explanted and either stored for (immuno‐)histology or polymerase chain reaction (PCR). For (immuno‐)histology, aortic rings from seven predefined regions (A, ascending aorta; B, aortic arch; C, proximal descending aorta; D, distal descending aorta; E, proximal abdominal aorta; F, suprarenal abdominal aorta; G, infrarenal abdominal aorta) (Figure 1D) were collected, rinsed with heparinized PBS and processed for further examination. For PCR, the remaining thoracic and abdominal aorta was stored at −80°C.

### 2.4 Blood plasma analysis

Blood plasma analysis was conducted after 3 (*n* = 15) or 6 months (*n* = 14), and serum levels of cholesterol, triglycerides, high density lipoprotein (HDL) and low density lipoprotein (LDL) cholesterol were analyzed, using standard assays designed for an automated clinical chemistry analyzer (series Cobas, Roche, Basel, Switzerland) at the Institute of Clinical Chemistry and Laboratory Diagnostics (Medical Faculty, Heinrich‐Heine‐University, Duesseldorf, Germany).

### 2.5 Histology

For histology, cryosections were used to perform the following staining procedures: hematoxylin–eosin (HE) staining, Movat's Pentachrome staining, von Kossa staining and Oil Red O staining. Image J (Wayne Rasband, National Institutes of Health, Bethesda, MD, USA) was used for morphometric analyses to detect intergroup differences. A previously published scoring system^12^ was utilized to quantify intima hyperplasia. In short, from each region of the aorta (regions A–G), one section divided into eight segments was analyzed to measure the mean intima‐to‐media ratio.

Calcification of the explanted aorta was quantified using a recently published scoring system.^13^ From each region of the aorta, a section was taken and divided into four segments, each of which was assessed using the scoring system. For the intima and media, the scoring range was 0–5. 0 = no calcification; 1 = microcalcification; 2 = mild calcification, brown discoloration; 3 = macrocalcification of <50% of the intima/media; 4 = macrocalcification of 50%–75% of the intima/media; 5 = macrocalcification of >75% of the intima/media, resulting in maximum values of 20.

### 2.6 Immunohistology

For immunohistology, cryosections were incubated at room temperature for 10 min with 0.25% Triton X‐100 and for 1 h with 5% bovine serum albumin +0.1% Tween‐20. Primary antibodies [anti‐alpha‐smooth muscle actin (αSMA) and anti‐CD3 (both Sigma‐Aldrich, Taufkirchen, Germany); anti‐CD68 (Abcam, Cambridge, UK)] were incubated with 1% bovine serum albumin and 0.1% Tween‐20 for 1 h at 37°C. Afterwards, secondary antibodies conjugated to the fluorophores Alexa488 and Alexa546 (Invitrogen, Carlsbad, CA, USA) were applied for 45 min in a dark and humid chamber at 37°C, following an incubation with 4′,6‐diamidino‐2‐phenylindole (DAPI) (Roth, Karlsruhe, Germany) for counterstaining. Finally, sections were covered with Leica medium (Leica Biosystems, Nussloch, Germany).

### 2.7 Quantitative RNA analysis

To analyze specific marker gene expressions in the explanted aorta, quantitative real‐time(RT)‐PCR was performed. RNA isolation was conducted using a commercially available kit (RNeasy Mini Kit, Qiagen, Hilden, Germany). In short, tissue was homogenized in TRIzol (Sigma‐Aldrich, Steinheim, Germany) and RNA was precipitated by isopropanol. RNA samples were collected after passing through a DNA‐removing column (Qiagen, Hilden, Germany) to perform quality analyses. Quantity and purity of the isolated RNA were defined spectrophotometrically (BioPhotometer plus; Eppendorf, Hamburg, Germany), and optical density values at 230, 260, and 280 nm were recorded. In addition, to identify the level of RNA degradation, the Agilent RNA 6000 Nano Kit (Agilent Technologies, Santa Clara, CA, USA) was used. Generation of cDNA was achieved by using QuantiTect Reverse Transcription Kit (Qiagen, Hilden, Germany). Quantitative RT‐PCR was conducted on a StepOnePlus cycler (Applied Biosystems, Foster City, CA, USA) using the Platinum SYBR Green PCR Master Mix (Invitrogen, Darmstadt, Germany) with a reaction volume of 20 μL: 50°C for 2 min, 95°C for 2 min, 95°C for 15 s and 60°C for 30 s (40 cycles), 95°C for 15 s, 60°C for 1 min, 95°C for 15 s and 60°C for 15 s.

The ΔΔCt method was used to determine the relative gene expression. Beta‐2 microglobulin (β_2_M) served as a house‐keeping gene. Primers for the following genes were analyzed and obtained from Invitrogen: bone morphogenetic protein‐2 (BMP2), interleukin 6 (IL‐6), osteopontin (OPN) and tumor necrosis factor alpha (TNF‐α). In Table 1, all primer sequences are displayed.

**TABLE 1:** Primer sequences for quantitative RT‐PCR.

Primer	Forward sequence	Reverse sequence
β_2_M	5′‐ATGAGTATTCCTGCCGGGTG‐3′	5′‐GGTATCCTCAGACCTCCATGC‐3′
BMP2	5′‐CTTTTGGTCACGATGGGAAGG‐3′	5′‐CCGCTGTTTGTGTTTCGCTT‐3′
IL‐6	5′‐ACGGTCAGAACACACCATCC‐3′	5′‐GTGTCCTAACGCTCATCTTCCT‐3′
OPN	5′‐TATTGATGAGGATGAGGACGATG‐3′	5′‐TGGTGAGAGTCATCGGGGT‐3′
TNF‐α	5′‐ACATCACCGAACCTCTGCTC‐3′	5′‐AGGAGTCTTTATTTCTCGCCAC‐3′

Abbreviations: BMP2, bone morphogenetic protein‐2; IL‐6, interleukin 6; OPN, osteopontin; TNF‐α, tumor necrosis factor alpha; β_2_M, beta‐2 microglobulin.

### 2.8 Statistics

Variables are presented as median and interquartile range (IQR). Group comparisons were executed by unpaired Student's *t*‐tests with or without Welch's correction or Mann–Whitney *U* tests, as indicated. *p* < 0.05 was assumed to indicate significance. GraphPad Prism 8 (GraphPad Software, San Diego, CA, USA) was used for data analysis.

## 3 RESULTS

### 3.1 Induction of hypercholesterolemia and hypertriglyceridemia

To induce a model of hypercholesterolemic and hypertriglyceridemic rabbits, animals were fed ad libitum with a pro‐atherosclerotic diet (*n* = 19). All rabbits showed a constant food intake (Figure S1A,B) and adequately gained weight (Figure S1C,D). No differences could be seen between group diet and group BI. Rabbits with a standard diet had an increased weight gain compared to rabbits receiving a pro‐atherosclerotic diet for 3 months (diet vs. control in g: 770 [IQR, 310–830] vs. 1130 [IQR, 1070–1365], *p* = 0.005; BI vs. control in g: 810 [IQR, 165–940] vs. 1130 [IQR, 1070–1365], *p* = 0.02) as well as for 6 months (diet vs. control in g: 830 [IQR, 370–1005] vs. 1400 [IQR, 1030–1570], *p* = 0.025; BI vs. control in g: 445 [IQR, 350–773] vs. 1400 [IQR, 1030–1570], *p* = 0.003).

**FIGURE 2:**
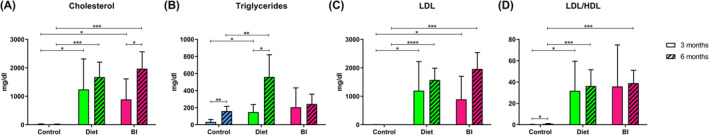
Blood plasma analysis. Cholesterol (A), triglyceride (B) and LDL (C) levels as well as the ratio of LDL to HDL (D) after 3 and 6 months. HDL, high density lipoprotein; LDL, low density lipoprotein. *p < 0.05; **p < 0.01; ***p < 0.001; ****p < 0.0001.

Groups diet and BI showed significantly elevated cholesterol levels after 3 months (diet vs. control: 682 mg/dl [IQR, 514–2250] vs. 17 [IQR, 16.5–27], *p* = 0.03; BI vs. control: 576 [IQR, 460–1480] vs. 17 [IQR, 16.5–27], *p* = 0.03) and 6 months (diet vs. control: 1470 mg/dL [IQR, 1260–2190] vs. 21 [IQR, 18–22.5], *p* = 0.0001; BI vs. control: 2030 [IQR, 1390–2490] vs. 21 [IQR, 18–22.5], *p* = 0.0001) compared to controls (Figure 2A). Triglyceride levels were significantly increased in group diet after 3 months (144 mg/dL [IQR, 69–228] vs. 19 [IQR, 18–53], *p* = 0.03) and 6 months (550 mg/dL [IQR, 315–810] vs. 143 [IQR, 110–216.5], *p* = 0.01) compared to controls (Figure 2B).

Also, LDL cholesterol levels were significantly higher in groups diet and BI after 3 months (diet vs. control: 682 mg/dL [IQR, 497–2160] vs. 4 [IQR, 4–5.5], *p* = 0.03; BI vs. control: 502 [IQR, 430–1556] vs. 4 [IQR, 4–5.5], *p* = 0.04) and 6 months (diet vs. control: 1460 mg/dL [IQR, 1205–2000] vs. 4 [IQR, 4–4], *p* < 0.0001; BI vs. control: 2020 [IQR, 1395–2465] vs. 4 [IQR, 4–4], *p* = 0.0001) compared to controls (Figure 2C), as were LDL to HDL cholesterol ratios after 3 months in group diet versus control (21.3 [IQR, 14.5–54.4] vs. 0.3 [IQR, 0.3–0.3], *p* = 0.03) and after 6 months in both groups compared to controls (diet vs. control: 41 [IQR, 20.3–50.0] vs. 0.8 [IQR, 0.7–1.2], *p* = 0.0008; BI vs. control: 42.3 [IQR, 26.6–48.2] vs. 0.8 [IQR, 0.7–1.2], *p* = 0.0002) (Figure 2D).

### 3.2 Inflammatory processes

**FIGURE 3:**
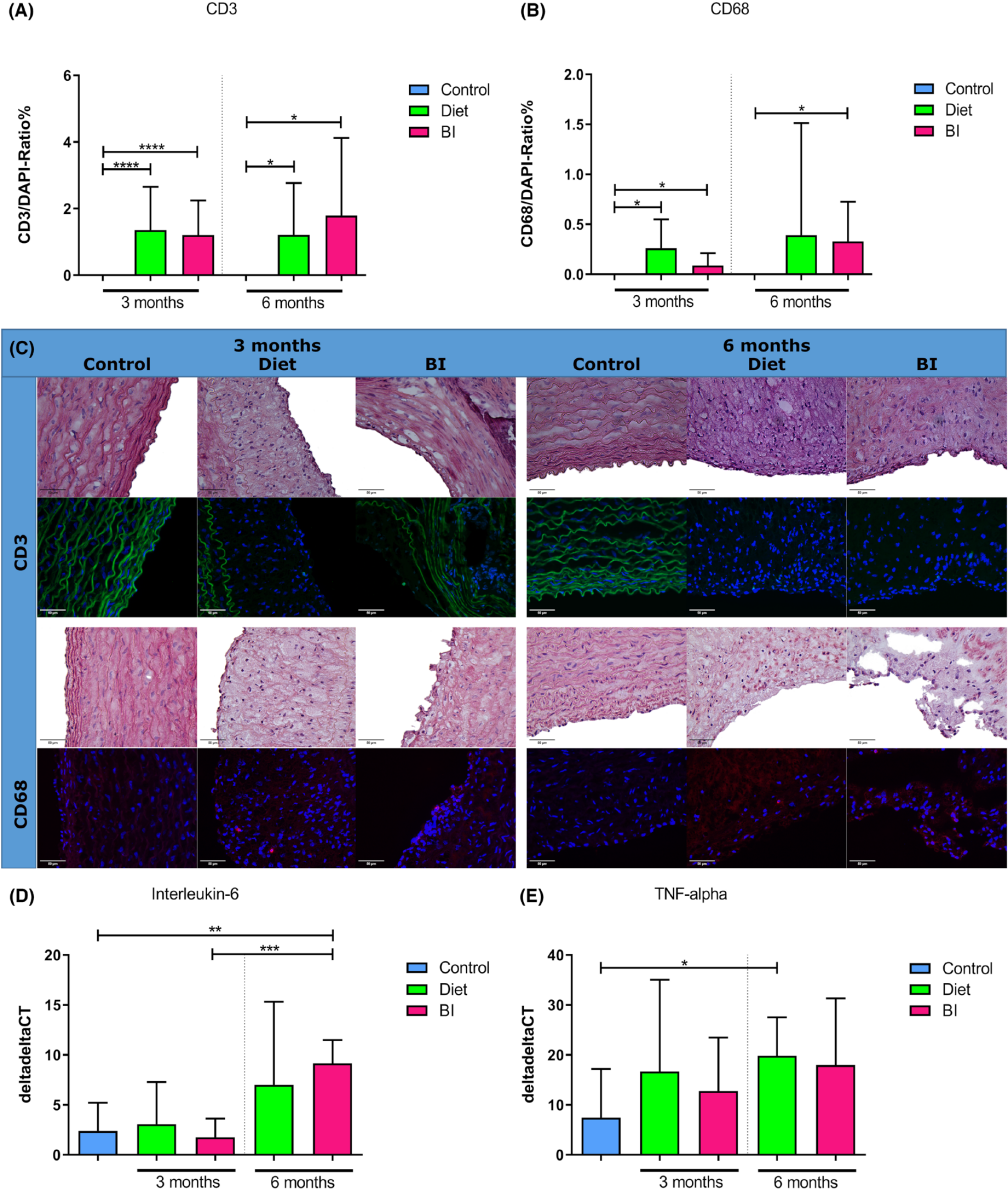
Inflammatory activity. (A), Immunohistology‐based ratio of CD3‐positive cells to all cells as a percentage after 3 and 6 months. (B) Ratio of CD68‐positive cells to all cells as a percentage after 3 and 6 months. (C) Representative CD3 (green) and CD68 (red) staining with matching HE stainings of controls, and groups diet and BI after 3 and 6 months. (D) IL‐6 gene expression after 3 and 6 months. (E) TNF‐alpha gene expression after 3 and 6 months. BI, balloon insufflation; IL‐6, Interleukin 6; TNF‐alpha, tumor necrosis factor alpha. *p < 0.05; **p < 0.01; ***p < 0.001; ****p < 0.0001.

Inflammatory activity in the aorta was assessed by immunohistology and quantitative PCR. After 3 months, the percentage of CD3‐positive cells was significantly increased in groups diet and BI compared to controls (in %, diet vs. control: 1.04 [IQR, 0.36–2.23] vs. 0 [IQR, 0–0], *p* < 0.0001; BI vs. control: 0.93 [IQR, 0.43–1.66] vs. 0 [IQR, 0–0], *p* < 0.0001) as well as after 6 months (in %, diet vs. control: 0.79 [IQR, 0.38–1.49] vs. 0 [IQR, 0–0], *p* = 0.02; BI vs. control: 1.24 [IQR, 0.39–2.18] vs. 0 [IQR, 0–0], *p* = 0.03) (Figure 3A,C). The percentage of CD68‐positive cells was significantly increased in groups diet and BI compared to controls after 3 months (in %, diet vs. control: 0.18 [IQR, 0–0.51] vs. 0 [IQR, 0–0], *p* = 0.01; BI vs. control: 0 [IQR, 0–0.19] vs. 0 [IQR, 0–0], *p* = 0.04) (Figure 3B,C). After 6 months, the percentage of CD68‐positive cells was significantly increased in group BI compared to controls (in %, 0.17 [IQR, 0–0.67] vs. 0 [IQR, 0–0], *p* = 0.02) (Figure 3B,C). IL‐6 gene expression tended to be enhanced in group diet compared to controls after 6 months (*p* = 0.2) and were significantly increased in group BI compared to controls (9.4 [IQR, 6.8–11.3] vs. 1.1 [IQR, 0.2–5.4], *p* = 0.004) (Figure 3D). TNF‐α gene expression tended to be enhanced in group diet vs. controls (*p* = 0.28) and in group BI vs. controls (*p* = 0.33) (Figure 3E). After 6 months, significantly increased TNF‐α gene was found in group diet compared to controls (17.3 [IQR, 13.1–28.0] vs. 0.9 [IQR, 0.1–20.6], *p* = 0.04) (Figure 3D). In group BI, TNF‐α only tended to increase as compared to controls (*p* = 0.16) (Figure 3E).

### 3.3 Intima hyperplasia in the aorta

**FIGURE 4:**
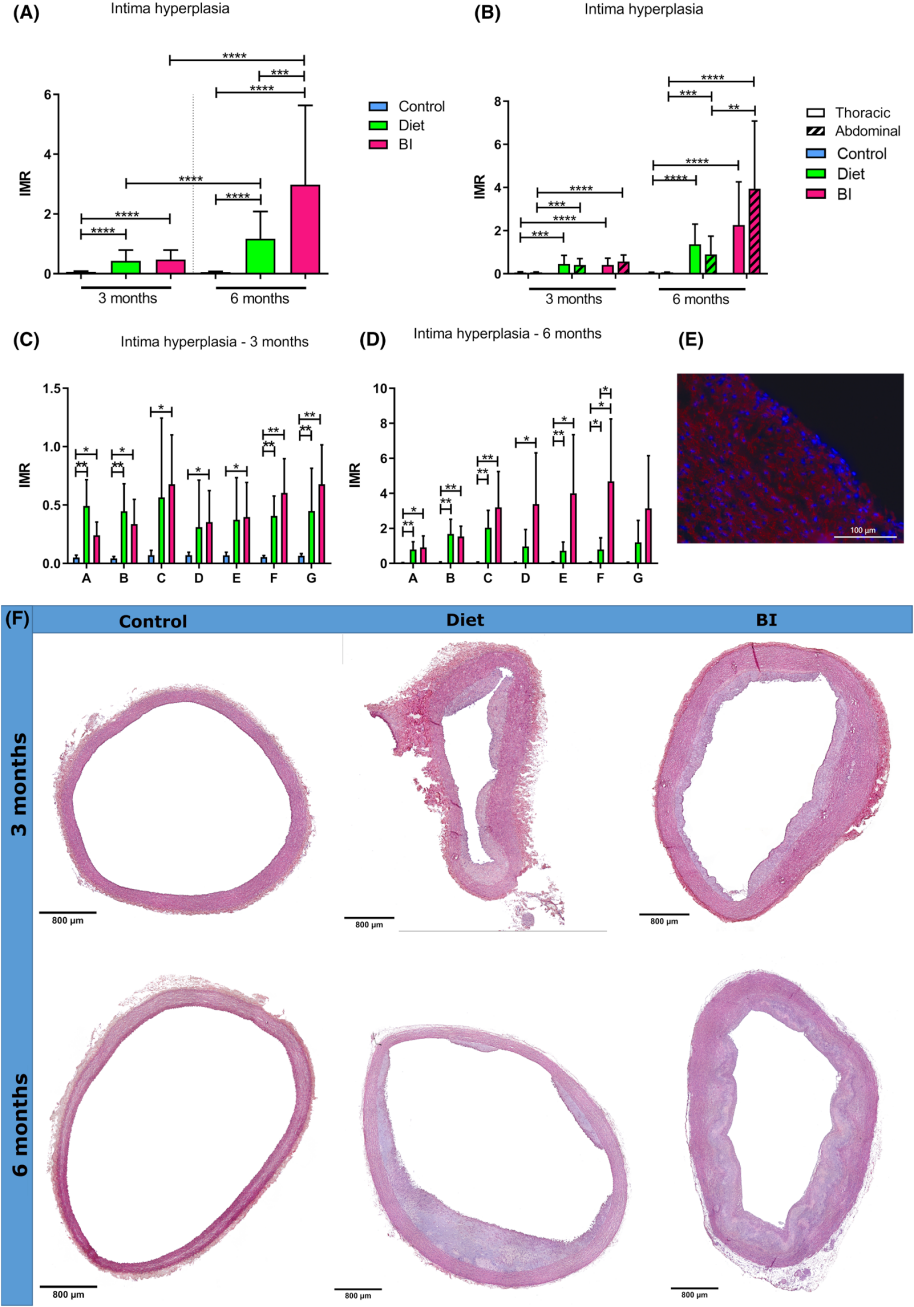
Aortic intima hyperplasia. (A) Intima‐media ratio of all regions together of group control, diet and BI after 3 and 6 months. (B–D) Intima‐media ratio of the thoracic and abdominal aorta after 3 and 6 months (B) as well as of predefined aortic regions after 3 months (C) and 6 months (D). (E) Representative αSMA staining after 3 months with αSMA‐positive cells (red) in the hyperplastic intimal region. (F) Representative HE stainings of the controls, group diet and BI after 3 and 6 months. BI, balloon insufflation; IMR, intima‐media ratio. *p < 0.05; **p < 0.01; ***p < 0.001; ****p < 0.0001.

After 3 and 6 months, significantly increased aortic intima hyperplasia was observed in groups diet and BI compared to controls in all regions together (after 3 months: diet vs. control 0.35 [IQR, 0.15–0.55] vs. 0.06 [IQR, 0.05–0.08], *p* < 0.0001; BI vs. control: 0.48 [IBR, 0.19–0.73] vs. 0.06 [IQR, 0.05–0.08], *p* < 0.0001; after 6 months: diet vs. control: 1.0 [IQR, 0.47–1.6] vs. 0.05 [IQR, 0.03–0.07], *p* < 0.0001; BI vs. control: 1.76 [IQR, 1.06–5.04] vs. 0.05 [IQR, 0.03–0.07], *p* < 0.0001) (Figure 4A,F), and also when analyzing separate regions after 3 (Figure 4C) and 6 months (Figure 4D). Intima hyperplasia in groups diet and BI was comparable after 3 months (*p* = 0.6) (Figure 4A,B), but after 6 months, intima hyperplasia was significantly higher in group BI compared to group diet in all regions together (*p* = 0.0003) (Figure 4A). However, when analyzing the thoracic and abdominal regions separately, significantly pronounced intima hyperplasia occurred only in the abdominal region in group BI when compared to group diet (*p* = 0.001) (Figure 4B). αSMA staining showed ubiquitous αSMA expression in the hyperplastic intimal regions and in the media (Figure 4E).

### 3.4 Atherosclerotic plaque formation

**FIGURE 5:**
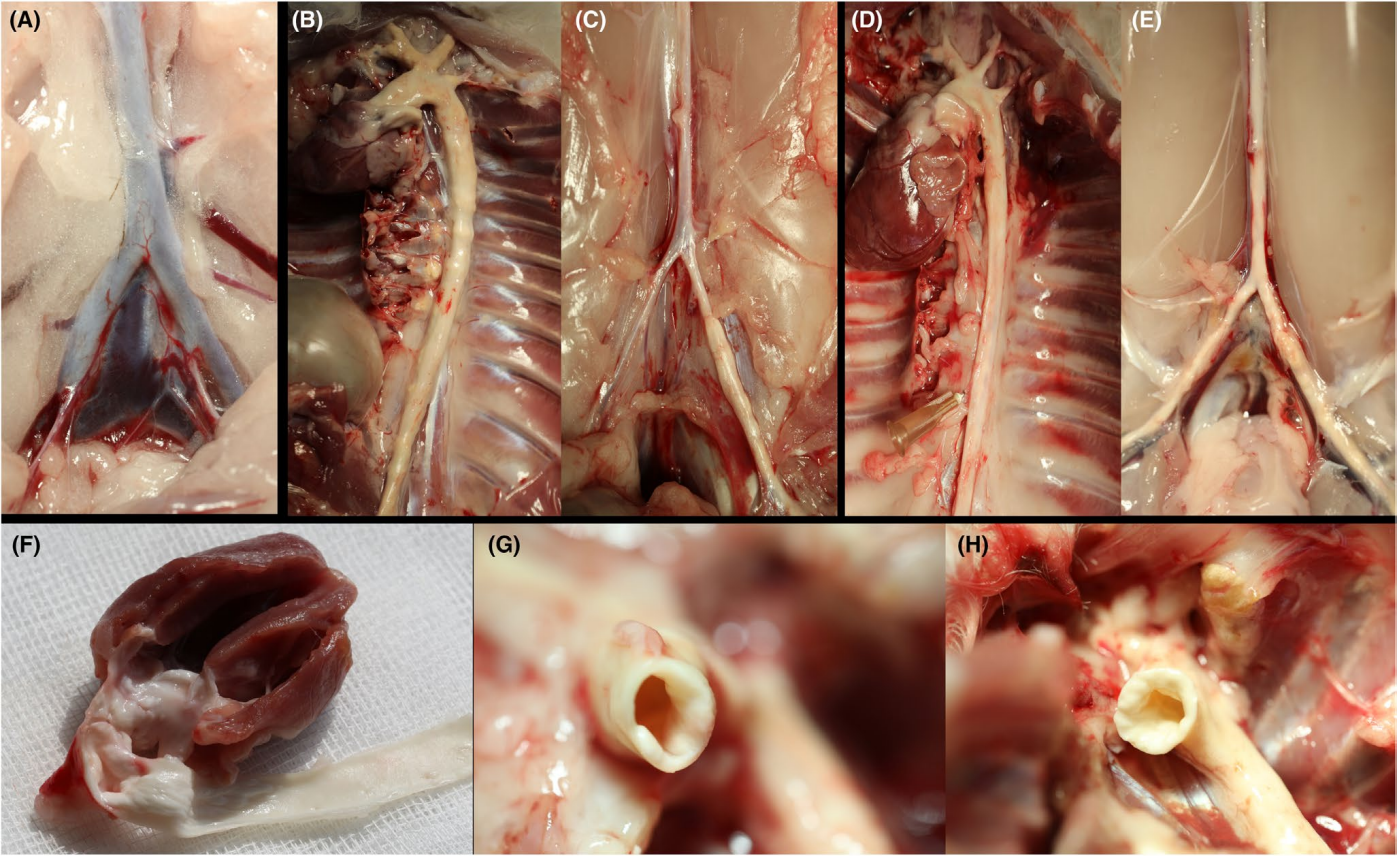
Macroscopic overview. (A) Infrarenal region of the aorta of group control after 6 months, (B) Thoracic aorta of group diet after 3 months, (C) Infrarenal region of the aorta of group diet after 3 months, (D) Thoracic aorta of group BI after 3 months, (E) Infrarenal region of the aorta of group BI after 3 months, (F) Atherosclerotic plaques in the ascending aorta of group diet after 3 months, (G) Lumen‐narrowing plaques in the ascending aorta of group diet after 3 months, (H) Lumen‐narrowing plaques in the ascending aorta of group BI after 3 months.

After 3 and 6 months, the external view presents an unaltered and non‐calcified aortic wall in group control (Figure 5A). In contrast, in groups diet and BI, the aortic wall was very thick and hard, with calcific irregularities along the whole aortic wall (Figure 5B–E). In groups diet and BI, we found ubiquitous lumen‐narrowing plaques (Figure 5F–H).

**FIGURE 6:**
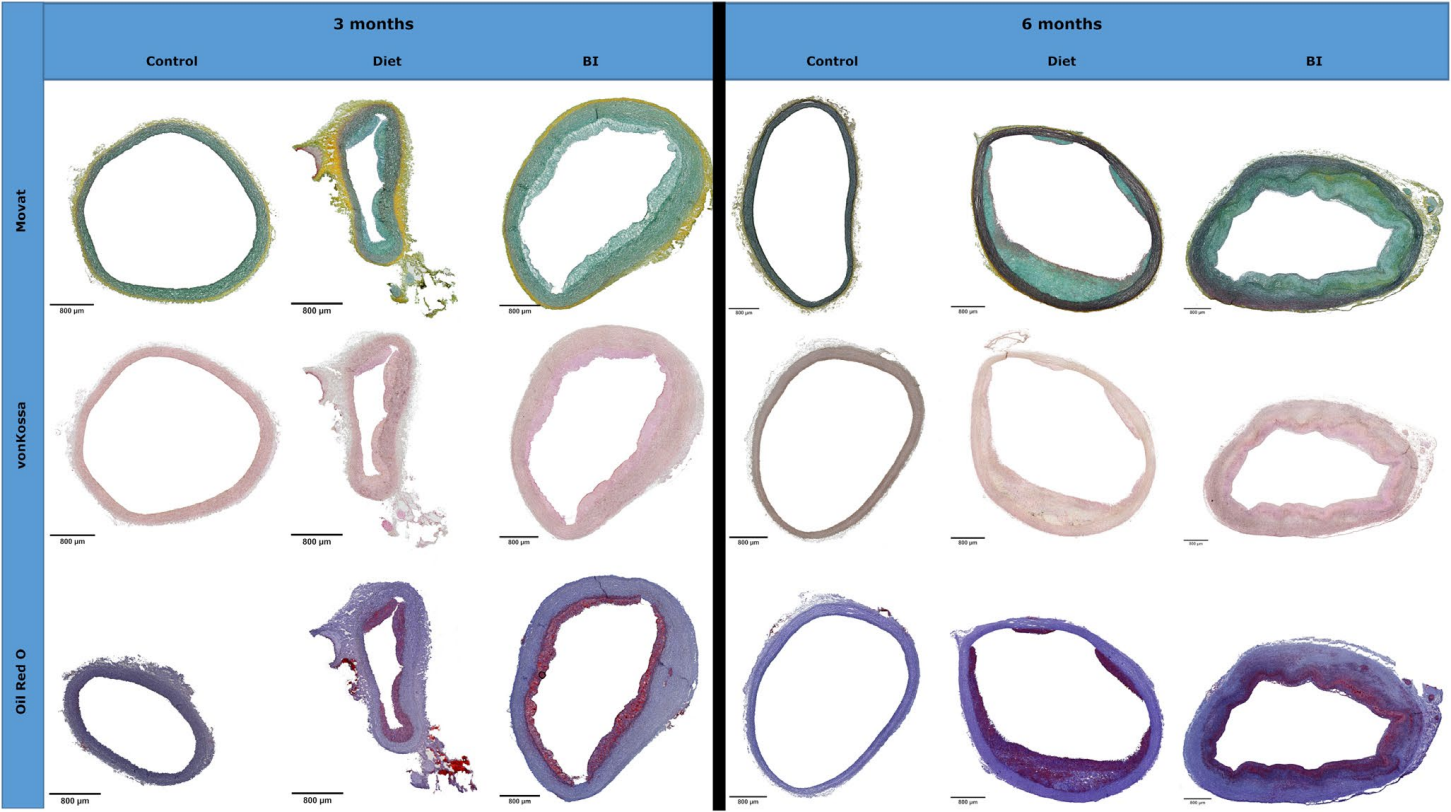
Histomorphological overview. Representative Movat Pentachrome, von Kossa and Oil Red O staining of groups control, diet and BI after 3 and 6 months. BI, balloon insufflation.

Movat Pentachrome staining was performed to focus on osteochondrogenic transformation. The detection of glycosaminoglycans shows the existence of chondrocytes and thus chondrogenic transformation. After 3 and 6 months, in contrast to controls, intensive green‐blue staining was observed in the hyperplastic regions of the intima as well as the media in groups diet and BI (Figure 6).

**FIGURE 7:**
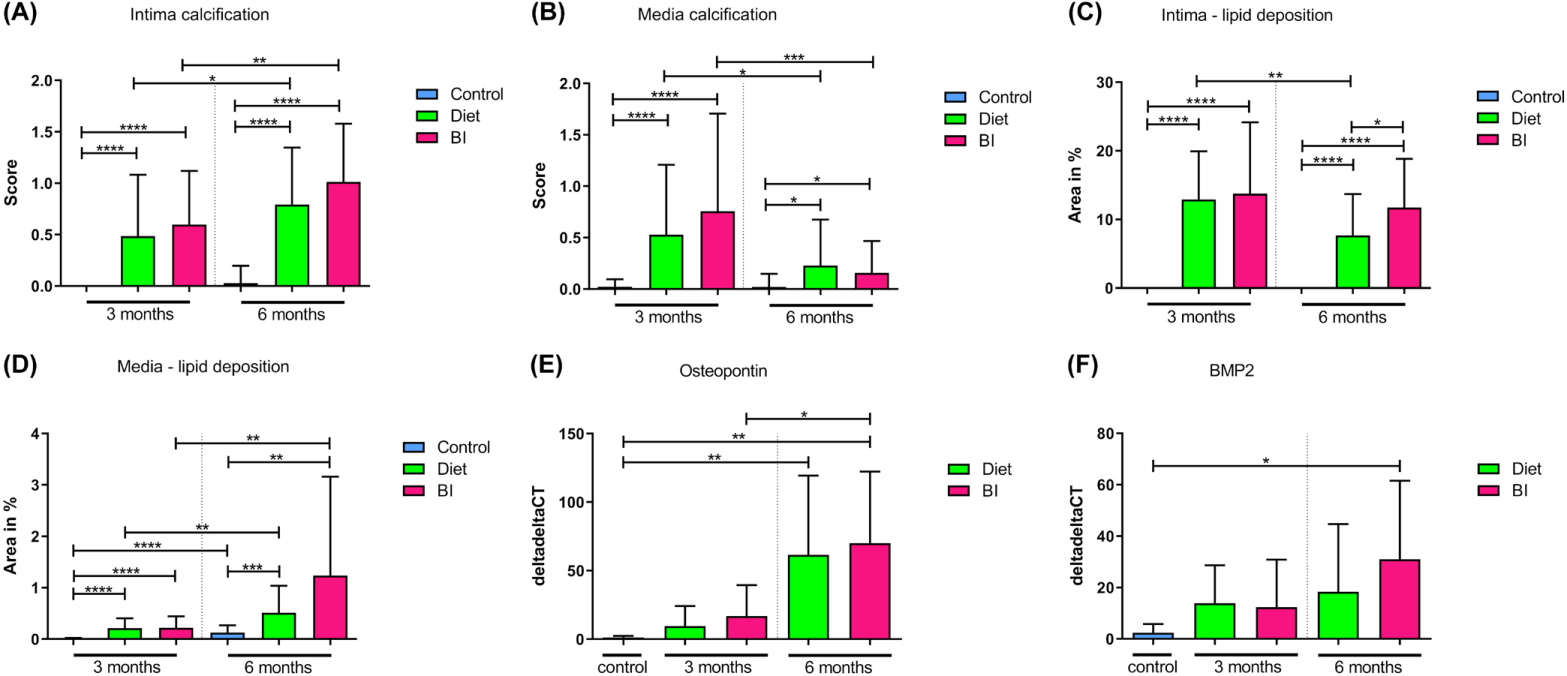
Calcifying and chondrogenic transformation. Intima (A) and media (B) calcification after 3 and 6 months as assessed by von Kossa staining. Lipid deposition in the intima (C) and media (D) after 3 and 6 months as assessed by Oil Red O staining. (E) OPN gene expression after 3 and 6 months. (F) BMP2 gene expression after 3 and 6 months. BI, balloon insufflation; BMP2, bone morphogenetic protein‐2; OPN, Osteopontin. *p < 0.05; **p < 0.01; ***p < 0.001; ****p < 0.0001.

Calcification was assessed by von Kossa staining (Figure 6). After 3 and 6 months, significantly increased intima calcification (Figure 7A) was seen in groups diet and BI compared to controls (after 3 months: diet vs. control 0.25 [IQR, 0–0.75] vs. 0 [IQR, 0–0], *p* < 0.0001; BI vs. control: 0.75 [IQR, 0–1.0] vs. 0 [IQR, 0–0], *p* < 0.0001; after 6 months: diet vs. control: 0.75 [IQR, 0.5–1.25] vs. 0 [IQR, 0–0], *p* < 0.0001; BI vs. control: 1.0 [IQR, 0.75–1.25] vs. 0 [IQR, 0–0], *p* < 0.0001). Also media calcification (Figure 7B) was significantly increased in groups diet and BI compared to controls after 3 months (diet vs. control: 0.25 [IQR, 0–1.0] vs. 0 [IQR, 0–0], *p* < 0.0001; BI vs. control: 0.5 [IQR, 0–1.5] vs. 0 [IQR, 0–0], *p* < 0.0001) and 6 months (diet vs. control: 0 [IQR, 0–0.5] vs. 0 [IQR, 0–0], *p* = 0.01; BI vs. control: 0 [IQR, 0–0.25] vs. 0 [IQR, 0–0], *p* = 0.02). Calcification in the intima was significantly increased in groups diet and BI after 6 months compared to 3 months, but no intergroup differences were seen.

Oil Red O staining was performed to demonstrate lipid accumulations (Figure 6). In group diet and BI, significantly increased lipid accumulations were found in the intima (Figure 7C) after 3 months (in %, diet vs. control: 11.7 [IQR, 8.0–19.3] vs. 0 [IQR, 0–0], *p* < 0.0001; BI vs. control: 11.5 [IQR, 4.9–21.3] vs. 0 [IQR, 0–0], *p* < 0.0001) and 6 months (in %, diet vs. control: 6.08 [IQR, 3.69–10.31] vs. 0 [IQR, 0–0], *p* < 0.0001; BI vs. control: 10.86 [IQR, 7.14–16.16] vs. 0 [IQR, 0–0], *p* < 0.0001) as well as in the media (Figure 7D) after 3 months (in %, diet vs. control: 0.15 [IQR, 0.08–0.28] vs. 0 [IQR, 0–0], *p* < 0.0001; BI vs. control: 0.17 [IQR, 00.6–0.34] vs. 0 [IQR, 0–0], *p* < 0.0001) and 6 months (in %, diet vs. control: 0.29 [IQR, 0.1–0.97] vs. 0.08 [IQR, 0.03–0.21], *p* = 0.0004; BI vs. control: 0.42 [IQR, 0.19–1.2] vs. 0.08 [IQR, 0.03–0.21], *p* = 0.003) compared to controls.

OPN gene expression tended to be elevated after 3 months in groups diet and BI compared to controls (diet vs. control: *p* = 0.1; BI vs. control: *p* = 0.052) (Figure 7E). After 6 months, significantly increased OPN gene expressions could be demonstrated in group diet and BI compared to controls (diet vs. control: 33.5 [IQR, 12.3–121.5] vs. 1.3 [IQR, 0.2–2.3], *p* = 0.006; BI vs. control: 64.9 [IQR, 31.3–81.9] vs. 1.3 [IQR, 0.2–2.3], *p* = 0.001) (Figure 7E). BMP2 levels were enhanced in groups diet and BI compared to controls after 3 months but the differences were not significant (diet vs. control: *p* = 0.0502; BI vs. control: *p* = 0.16). After 6 months, significantly elevated BMP2 levels could be seen in group BI compared to controls (22.3 [IQR, 14.5–46.9] vs. 1.2 [IQR, 0.3–4.4], *p* = 0.02) (Figure 7F).

## 4 DISCUSSION

In the present study, a rabbit model exhibiting macroscopic atherosclerotic plaques, thereby allowing research on vascular hemodynamics and the influence of ECC in particular, was developed and characterized in detail.

This project specifically aimed to mimic the pathophysiological mechanisms involved in atherosclerosis development and progression. Atherosclerosis is a multifocal, immunoinflammatory disease of medium‐sized and large arteries fueled by lipids.^14^ A multitude of risk factors contribute to the development of atherosclerosis, among which an elevated plasma cholesterol level is probably unique in being sufficient to advance the development of atherosclerosis even in the absence of additional risk factors.^15^ In the present study, rabbits fed with a pro‐atherosclerotic diet, including 0.3% cholesterol, showed excessively elevated plasma cholesterol levels. LDLs have an essential role as vehicles to deliver cholesterol to peripheral tissues, while increased LDL levels are associated with an elevated risk of cardiovascular disease.^15^ In this study, rabbits on a pro‐atherosclerotic diet showed significantly increased LDL cholesterol levels as well as an increased LDL to HDL cholesterol ratio compared to controls on standard chow.

Rabbits are considered to exhibit a similar lipoprotein metabolism to humans, and unlike other common laboratory species such as mice, in which HDL is the predominant plasma lipoprotein, rabbits transport cholesterol predominantly via apolipoprotein B‐containing particles such as LDL and VLDL (very low‐density lipoprotein). Thus, rabbits have been extraordinarily useful in experimental research to illustrate the role of elevated plasma cholesterol in the initiation of atherosclerosis.^16^

Beyond the cholesterol burden, rabbits on a pro‐atherosclerotic diet showed elevated triglyceride levels compared to controls. Several studies support the concept that elevated triglyceride levels are related to an increased risk of cardiovascular disease caused by atherosclerosis.^17^, ^18^, ^19^, ^20^ Nordestgaard et al. demonstrated that elevated triglyceride‐rich lipoproteins are associated with atherosclerosis, and showed evidence that high concentrations of triglyceride‐rich lipoproteins cause low‐grade inflammation resulting in increased triglyceride‐rich lipoprotein uptake into macrophage foam cells in the arterial intima.^18^ Although the recruitment of monocytes into the arterial wall with subsequent differentiation into macrophages initially has a protective function as they remove cytotoxic and proinflammatory oxidized LDL as well as apoptotic cells, an intensive accumulation of macrophages and their continuous uptake of oxidative LDL is considered to finally lead to the development of atherosclerotic lesions.^15^, ^21^, ^22^ In line with these previous data, we demonstrated significantly increased macrophage infiltration in rabbits under a pro‐atherosclerotic diet with and without an additional balloon insufflation compared to controls on standard chow. Interestingly, macrophage‐rich lesions have been shown to undergo plaque rupture and induce acute clinical complications of atherosclerosis, such as myocardial infarction or stroke.^23^ Pro‐inflammatory macrophages in particular initiate and sustain tissue inflammation by producing inflammatory cytokines such as IL‐6 or TNF‐α,^24^ among which IL‐6 particularly has been reported to be associated with enhanced inflammation in the context of atherosclerosis.^25^ Mimicking these pro‐inflammatory mechanisms, significantly elevated IL‐6 and TNF‐α gene expression were measured in our animal model.

Furthermore, recent studies have drawn the attention to the role of T‐cells as critical promoters in the pathogenesis of atherosclerosis.^26^, ^27^, ^28^ In this context, among other factors that serve as T cell‐activating antigens, LDL and its core protein apolipoprotein B show strong association with atherosclerosis.^29^ These findings support the proposal that LDL is a significant self‐antigen that drives an autoimmune response in atherosclerotic plaques.^26^ Taken together, these observations suggest that T‐cells are involved in the initiation, progression, regression and ultimately rupture of atherosclerotic plaques.^26^ In the present study, relevant T‐cell infiltration is demonstrated in rabbits with pro‐atherosclerotic diet.

Remarkable intima hyperplasia was induced in all rabbits under a pro‐degenerative diet with and without additional balloon insufflation injury, especially in the thoracic aorta. In the abdominal aorta, intima hyperplasia was pronounced in rabbits with additional aortic wall injury compared to rabbits under a pro‐degenerative diet alone. In this context, several studies have shown that atherosclerotic lesions predominantly develop at vascular sites of preexisting intimal thickening or intima hyperplasia.^30^, ^31^, ^32^, ^33^ Subbotin et al. demonstrated that excessive intima hyperplasia occurred prior to lipid depositions, resulting in the initiation of atherosclerosis.^33^ In our animal model, extensive lipid depositions were found in rabbits on a pro‐atherosclerotic diet independently of additional aortic ballon injury. Such intracellular lipid accumulations or foam cell formations are typical of early but also late atherosclerotic plaques which predominantly contain large amounts of cholesterol esters.^14^, ^15^ The excess of lipid uptake by macrophages perpetuates the inflammatory response, and oxidized LDL induces specific cascades which maintain endothelial cell activation, monocyte recruitment and foam cell formation—resulting in a vicious circle.^34^ Therefore, cholesterol accumulation is considered as a “hallmark of atherosclerotic lesions”.^35^

As a consequence of pro‐degenerative processes, significant intima and media calcification occurred in group diet as well as in group BI. Atheroma plaque calcification is a central step in advanced atherosclerosis, promoted by osteogenic transdifferentiation.^36^, ^37^ In particular, pericytes and vascular smooth muscle cells differentiate into osteoblast‐like phenotypes generating a mineralized matrix incorporating calcium deposits, as also occurs in bone tissue formation.^38^, ^39^ The resulting microcalcification represents an early stage of the vascular calcification cascade in both the intima and media.^37^, ^40^ Typically associated markers of osteogenic transformation are OPN and BMP2, the gene expression levels of which were elevated in rabbits under a pro‐atherosclerotic diet in our model.

Taken together, our developed atherosclerotic animal model can be used for research on vascular hemodynamics and the influence of ECC in atherosclerotic animals in particular. Until now, only computational fluid dynamics could be used to demonstrate blood flow profiles in vessel geometries, turbulences, blood flow velocities and wall shear stress.^41^, ^42^ To validate such computational models and to assess the impact of blood flow or wall shear stress, magnetic resonance imaging during ECC in atherosclerotic animals is required. This is particularly important, as most of the patients requiring ECC treatment suffer from atherosclerosis, which predisposes to embolic organ ischemia including stroke. The combination of our published miniaturized ECC model^7^ with the atherosclerosis model presented here can help us to improve our understanding of the pathophysiology of ECC‐related blood flow impairments and thus to develop strategies for avoiding perfusion‐associated organ damage.

Beyond the development and profound characterization of a diet‐induced atherosclerotic rabbit model, we specifically evaluated the need for additional ballon injury of the vascular intima in promoting atherosclerosis initiation and progression. Based on our findings, additional intraaortic ballon insufflation, as previously published,^43^ does not seem to be necessary, as we only found enhanced intima hyperplasia in the abdominal region without enhanced lipid deposition or calcification. Thus, to minimize surgical trauma in animals and therefore minimize pain and stress, an additional intraaortic ballon insufflation should be avoided.

A major limitation of our study in terms of translation is the fact that rabbits and humans exhibit different hemodynamics, which also play a role in vascular disease development as well as the occurrence of subsequent complications. However, metabolic similarities between humans and rabbits, particularly in lipid metabolism resulting in predisposition to plaque formation, make rabbit models very useful in atherosclerosis research, especially in the context of revealing the basic mechanisms contributing to health impairment.

## 5 CONCLUSIONS

The present study provides the design and detailed characterization of a model of diet‐induced atherosclerosis in rabbits. Multiple pathophysiological mechanisms, as well as a high and ubiquitous arterial plaque burden, mimic the human atherosclerotic disease. An additional intraaortic ballon insufflation injury results in enhanced intima hyperplasia especially in the abdominal region, however, it does not lead to pronounced lipid deposition or calcification, which would contribute to plaque formation. Thus, an additional injury of the intima by an intraaortical ballon insufflation does not appear to be absolutely necessary if the focus of a study is on calcification and plaque formation, so the additional surgical intervention can be avoided.

The developed animal model is suitable for use in future studies on the interaction between atherosclerotic plaques and arterial blood flow, and hemodynamic changes due to ECC in particular.

## AUTHOR CONTRIBUTIONS

**Anna Kathrin Assmann:** Conceptualization; data curation; funding acquisition; visualization; writing – original draft. **Jan Buschmann:** Data curation. **Sinje Reimers:** Data curation. **Aleyna Karakas:** Data curation. **Elvira Weber:** Writing – review and editing. **Hug Aubin:** Writing – review and editing. **Artur Lichtenberg:** Writing – review and editing. **Alexander Assmann:** Conceptualization; formal analysis; supervision; writing – review and editing.

## FUNDING INFORMATION

Supported by the German Heart Foundation/German Foundation of Heart Research.

## CONFLICT OF INTEREST STATEMENT

Nothing to declare.

## ETHICS APPROVAL STATEMENT

Every experiment was conducted according to the “Guide for the Care and Use of Laboratory Animals” and approved by the state animal care committee (reference number 81‐02.04.2020.A383).

## Supporting information


**Figure S1.**.

## Data Availability

The authors confirm that the data supporting the findings of this study are available within the article and its supporting information.
