# Supplementary figures and images for: Development of an atherosclerosis rabbit model to evaluate the hemodynamic impact of extracorporeal circulation
Source: Animal Model Exp Med. 2025 Feb 5;8(3):523–33. doi: 10.1002/ame2.12556 (PMC11904102; doi:10.1002/ame2.12556)

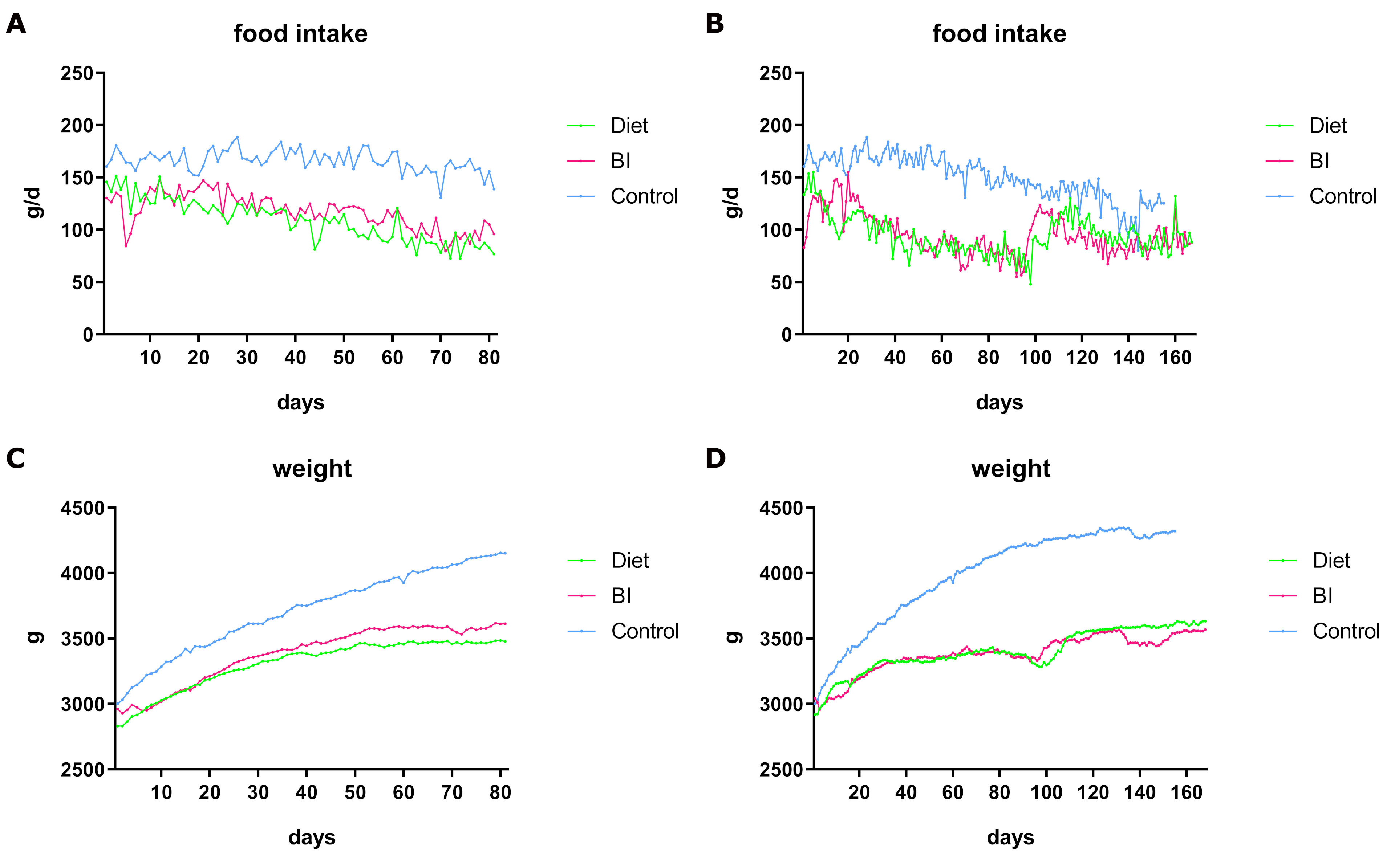

Supplement: Supplementary file 1 — Figure S1.. [file AME2-8-523-s001.jpg]
